# Quantification of Fecal Short Chain Fatty Acids by Liquid Chromatography Tandem Mass Spectrometry—Investigation of Pre-Analytic Stability

**DOI:** 10.3390/biom9040121

**Published:** 2019-03-28

**Authors:** Gerhard Liebisch, Josef Ecker, Sebastian Roth, Sabine Schweizer, Veronika Öttl, Hans-Frieder Schött, Hongsup Yoon, Dirk Haller, Ernst Holler, Ralph Burkhardt, Silke Matysik

**Affiliations:** 1 Institute for Clinical Chemistry and Laboratory Medicine, University Hospital Regensburg, Franz-Josef-Strauss-Allee 11, 93053 Regensburg, Germany; sebastian.roth@ukr.de (S.R.); Schweizer.sabine@gmx.net (S.S.); Veronika.Oettl@stud.uni-regensburg.de (V.Ö.); hans-frieder.schoett@isas.de (H.-F.S.); Ralph.Burkhardt@ukr.de (R.B.); silke.matysik@ukr.de (S.M.); 2 Nutritional Physiology, Technical University of Munich, 85354 Freising, Germany; josef.ecker@tum.de; 3 ZIEL-Institute for Food & Health, Technical University of Munich, 85354 Freising, Germany; Hongsup.Yoon@tum.de (H.Y.); dirk.haller@tum.de (D.H.); 4 Chair of Nutrition and Immunology, Technical University of Munich, 85354 Freising, Germany; 5 Department of Hematology and Oncology, Internal Medicine III, University Hospital Regensburg, 93053 Regensburg, Germany; Ernst.Holler@ukr.de

**Keywords:** SCFA, mass spectrometry, feces, LC-MS/MS, method validation

## Abstract

Short chain fatty acids (SCFAs) are generated by the degradation and fermentation of complex carbohydrates, (i.e., dietary fiber) by the gut microbiota relevant for microbe–host communication. Here, we present a method for the quantification of SCFAs in fecal samples by liquid chromatography tandem mass spectrometry (LC-MS/MS) upon derivatization to 3-nitrophenylhydrazones (3NPH). The method includes acetate, propionate, butyrate, and isobutyrate with a run time of 4 min. The reproducible (coefficients of variation (CV) below 10%) quantification of SCFAs in human fecal samples was achieved by the application of stable isotope labelled internal standards. The specificity was demonstrated by the introduction of a quantifier and qualifier ions. The method was applied to investigate the pre-analytic stability of SCFAs in human feces. Concentrations of SCFA may change substantially within hours; the degree and kinetics of these changes revealed huge differences between the donors. The fecal SCFA level could be preserved by the addition of organic solvents like isopropanol. An analysis of the colon content of mice either treated with antibiotics or fed with a diet containing a non-degradable and -fermentable fiber source showed decreased SCFA concentrations. In summary, this fast and reproducible method for the quantification of SCFA in fecal samples provides a valuable tool for both basic research and large-scale studies.

## 1. Introduction

Short-chain fatty acids (SCFAs) acetate (FA 2:0; annotation according to [1]), propionate (FA 3:0), and butyrate (FA 4:0) are generated as end products, by the degradation and fermentation of indigestible carbohydrates by the gut microbiota, a process termed saccharolytic fermentation [2]. SCFAs reach circulation via the portal vein and can then alter the host metabolism and physiology substantially. They can act as signaling molecules as ligands of G-protein-coupled receptors [3]. Physiologically, SCFAs are implicated in the increase of anorexic hormone production and energy expenditure [3]. Consequently, SCFA production was linked to preventing the progression of obesity and related complications, such as type 2 diabetes mellitus and nonalcoholic fatty liver disease (NAFLD) [3,4]. Related to these functions, we have just recently identified in mice that gut microbiota-derived acetate is an important precursor for the synthesis of fatty acids and phospholipids in the liver [5].

Branched chain fatty acids (BCFA) like isobutyrate (FA 3:0(2Me)), 2-methylbutyrate (FA 4:0(2Me)), and isovalerate (FA 4:0(3Me)) are derived from the fermentation of branched-chain amino acids (BCAAs) [6]. In contrast to straight chain SCFA, these compounds are considered detrimental to colonic and metabolic health [4].

SCFAs are typically quantified by gas chromatography (GC), liquid chromatography (LC), nuclear magnetic resonance (NMR), and capillary electrophoresis (CE) (recently reviewed in the literature [7]). In the last years, LC coupled to tandem mass spectrometry (LC-MS/MS) was increasingly applied to quantify SCFA. These methods require derivatization using, for example, 2-nitrophenylhydrazin [8], 3-nitrophenylhydrazine [9], O-benzylhydroxylamine [10], or aniline [11].

Here, we present a novel LC-MS/MS method for the quantification of major SCFAs in fecal samples, based on 3-nitrophenylhydrazone (3NPH) derivatization. The fast and reproducible method was applied to investigate the pre-analytic stability of SCFA in human feces. Furthermore, the colon contents were analyzed from mice treated with antibiotics to eliminate microbial SCFA producers, or fed a diet containing solely a non-degradable and -fermentable fiber source (i.e., cellulose).

## 2. Materials and Methods 

### 2.1. Reagents

Formic acid of analytical grade, acetonitrile, and isopropanol LiChrosolv were purchased from Merck (Darmstadt, Germany). Acetic, propionic, butyric and isobutyric acid, pyridine, *N*,*N-*dimethylglycine (DMG), and *N*-(3-dimethylaminopropyl)-*N*′-ethylcarbodiimide (EDC) were purchased from Sigma Aldrich Chemie GmbH (Steinheim, Germany). The methanol LC-MS Chromasolv was purchased from Honeywell Riedel-de Haën (Seelze, Germany). [^13^C,D_3_]-acetic acid, [D_5_]-propionic acid, and [D_7_]-butyric acid were from Cambridge Isotope Laboratories (Tewksbury, MA, USA). Stock solutions of the SCFAs, including the labeled compounds, were prepared in water and were stored at −80 °C. 

### 2.2. Preparation of Human Fecal and Murine Colon Content Homogenates 

The human fecal samples for the method development were obtained from healthy volunteers and were homogenized as described previously [12]. In brief, the fecal samples were homogenized in a gentleMACS™ Dissociator (Miltenyi Biotec GmbH, Bergisch Gladbach, Germany) in 70%-isopropanol. The dry weight (DW) was determined by the overnight drying of an aliquot in a vacuum centrifuge. The feces homogenates were diluted to a concentration of 2.0 mg DW/mL. The samples were stored at −80 °C and were kept on ice during processing. For the SCFAs, an aliquot of the homogenate was centrifuged and the clear supernatant was subjected to derivatization.

The colon content from the mice were homogenized by bead beating (1.4 mm ceramic beads) using a Precellys homogenizer (Bertin Technologies, Montigny Le Bretonneux, France). The samples (10 to 30 mg weight) were homogenized in 1 mL 70%-isopropanol twice with 15 s agitation and 60 s break. Then, 300 µL of the homogenate was used to determine the DW. The dilution and further handling were identical as that described for the human samples.

### 2.3. Sample Preparation

Clear supernatants of the fecal and colon samples were subjected to 3-nitrophenylhydrazone (3NPH) derivatization according to the method described by Han et al. [9], with some modifications. In brief, 50 µL of an aqueous internal standard mixture containing 100 µg/mL each of [^13^C,D_3_]-acetic acid and [D_5_]-propionic acid, and 500 µg/mL of [D_7_]-butyric acid were added to 50 µL supernatant of feces homogenate (2 mg DW/mL) and mixed. Then, 20 µL of 200 mM 3-nitrophenylhydrazine hydrochloride and 20 µL of 120 mM *N*-(3-dimethylaminopropyl)-*N*′-ethylcarbodiimide hydrochloride were added and mixed for 30 min at 40 °C. The reaction was quenched by the addition of 200 µL of 0.1% formic acid and were used for the LC-MS/MS analysis. 

### 2.4. Calibrators and Quality Controls

Pooled human fecal homogenates were used to prepare the matrix calibrators [12]. For low levels (level II and III), this pool was diluted with 70%-isopropanol. High levels (level V and VI) were generated by the supplementation of SCFA from concentrated aqueous solutions. The calibration lines contained six levels in the concentration range, shown in Figure S1A–D. 

The samples from three individuals with low, medium, and high SCFAs were aliquoted and used as the quality control samples and for the method validation (see Section 3.4).

### 2.5. LC-MS/MS

SCFAs quantification was performed by liquid chromatography-tandem mass spectrometry (LC-MS/MS). A 1200 series binary pump, isocratic pump, and degasser (Agilent, Waldbronn, Germany) with an HTC Pal autosampler (CTC Analytics, Zwingen, Switzerland) was coupled to a hybrid triple quadrupole linear ion trap mass spectrometer API 4000 Q-Trap equipped with a Turbo V source ion spray (Applied Biosystems, Darmstadt, Germany). 

The SCFAs were separated using a Kinetex® 2.6 µm XB-C18, 50 × 2.1 mm (Phenomenex, Torrance, CA, USA), with water as mobile phase A and acetonitrile as mobile phase B, both containing 0.1% formic acid. The gradient elution started with 10% B, with a linear increase to 20% B at 0.3 min, followed by an increase to 23% B at 2.5 min. The column was cleaned with 100% B from 2.6 to 3.0 min, and was re-equilibrated from 3.1 to 4 min with 90% A. The column flow was 500 µL at 60 °C and 2 µL of the sample were injected. To minimize the contamination of the mass spectrometer, the column flow was only directed from 55 to 160 s into the mass spectrometer, using a diverter valve. Otherwise, methanol with a flow rate of 250 µL/min was delivered into the mass spectrometer.

The Turbo Ion Spray source was operated in the negative ionization mode, using the following settings: ion spray voltage = −4500 V, ion source heater temperature = 450 °C, source gas 1 = 55 psi, source gas 2 = 50 psi, and curtain gas setting = 25 psi. The analytes were monitored in the multiple reaction monitoring (MRM) mode, and the mass transitions and MS parameters are shown in Table 1. Quadrupoles Q1 and Q3 were operated at unit resolution.

### 2.6. Method Validation

The method validation was performed on the basis of the FDA [13] and EMA [14] guidelines on bioanalytical method validation.

The limit of the limit of detection (LoD) was determined by calculation of the signal to noise (S/N) ratios, as the concentration with S/N = 3 for the analytes devoid of chemical background. For SCFAs with a chemical background in the blank samples, the LoD was calculated from a limit of blank (LoB) and a variation of a sample with a low concentration, as described previously [15] (Supplementary Table S1), as follows:

(1) LoB=mean(blank)+1.645 SD(blank)

(2) LoD=LoB+1.645 SD(low concentration sample)

### 2.7. Investigation of Pre-Analytic Stability

Three healthy volunteers collected fresh stool samples and transported them to the laboratory on ice. The samples were homogenized in water using a gentleMACS™ Dissociator, as described above. The fecal homogenates were aliquoted and diluted with the same volume of water, methanol, or isopropanol, to receive 70% organic solutions for alcohol containing homogenates. The aliquots of these homogenates were frozen either immediately at −80 °C, or were stored for 0.5, 1, 3, 6, 24, 72, and 168 h at room temperature or 4 °C before freezing.

### 2.8. Mouse Experiments

Specific pathogen free (SPF) C57BL/6 N mice were housed at 22  ±  1 °C and 50%–60% relative humidity, with a 12  h light–dark cycle as described previously in detail [5]. The mice were fed a chow diet (autoclaved, V1534, ssniff Spezialdiäten GmbH, Soest, Germany) ad libitum. For the antibiotic treatment, sole mice were fed a chow mash containing vancomycin (0.25 g/L) and metronidazol (1 g/L) (Sigma Aldrich, Steinheim, Germany) ad libitum for two days at the age of eight weeks, without any additional food (per group: male/female, 1/1). The control group received the same diet supplemented with water. The mouse experiment was performed according to the relevant ethical guidelines. The breeding and experimental use of the mice in the facilities at the Technische Universität München (School of Life Sciences Weihenstephan) was approved by the local institution in charge (Regierung von Oberbayern; approval number 55.2-1-54-2531-99-13 and 55.2-1-54-2532-17-2015). 

For dietary intervention, the C57BL/6 N mice at the age of six weeks were fed for two weeks either a standard chow diet (5% grain–soybean-based crude fiber extract; V1534, ssniff) or a purified control diet with a comparable carbohydrate, fat, and protein content as chow, but with purified cellulose (5%) as the sole fiber source (autoclaved, S5745-E702, ssniff).

### 2.9. Statistical Analyses

IBM SPSS Statistics 25 was used for the statistical testing. The mouse experiments were analyzed using a non-parametric Mann–Whitney U test.

## 3. Results

### 3.1. Sample Preparation and Chromatography

The aim of the current study was to develop an accurate, robust, and fast method for the quantification of SCFA (up to four C-atoms) in fecal samples by LC-MS/MS. The SCFAs were determined in an aliquot representing 100 µg DW. The derivatization of SCFAs to 3-nitrophenylhydrazones (3NPH) was performed according to the protocol described by Han et al. [9]. The derivatization reaction was stopped by the addition of formic acid. For accurate quantification, stable isotope labeled SCFAs were added to the derivatization as internal standards (ISs) for each straight chain SCFA (i.e., [^13^C,D_3_]-FA 2:0, [D_5_]-FA 3:0, [D_7_]-FA 4:0).

**Figure 1:**
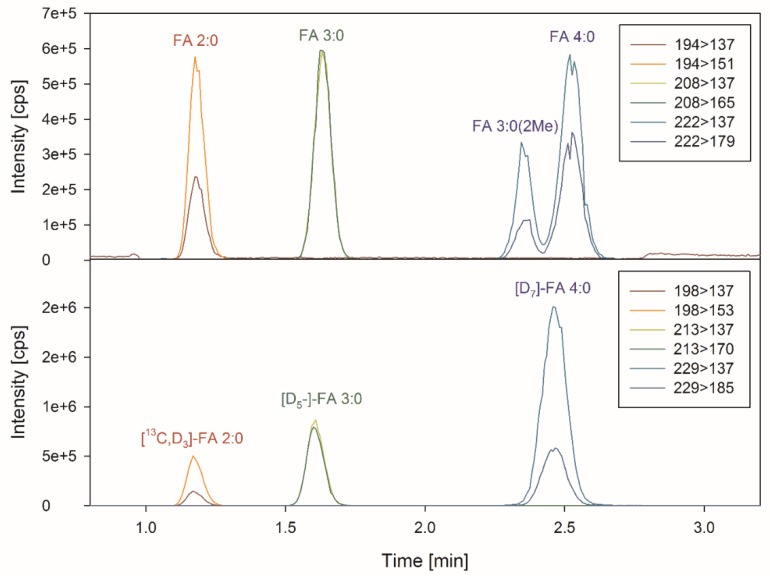
Chromatogram of short chain fatty acids (SCFAs) and their internal standards. Displayed are the extracted ion chromatograms of SCFAs, and their internal standards of a representative human fecal sample.

The chromatographic separation of SCFA–3NPHs was optimized in order to achieve the fast separation of isomeric butyric acid FA 4:0 and iso-butyric acid FA 3:0(2Me). A water–acetonitrile gradient was applied on a C18 core shell material. FA 4:0 and FA 3:0(2Me) eluted at 2.36 and 2.53 min showed an acceptable separation with a peak resolution R ≈ 1.4 (Figure 1), respectively. Including column cleaning and re-equilibration, the method has a run time of 4 min. 

### 3.2. Specificity and Matrix Effects

Here, we applied, in addition to the fragment ion m/z 137 resulting from the 3NPH-moiety of the derivatives, a fragment specific for the respective SCFAs generated most likely from a neutral loss of isocyanic acid (Table 1; Supplementary Figure S1A–D for the product ion spectra). As a result of their lower background level, neutral loss fragment ions were used as quantifier. The calculation of the quantifier/qualifier ion ratios permits the evaluation of specificity for individual samples. The quantifier/qualifier ratios calculated for a batch with more than 100 different human fecal samples (data not included here) showed the following variations: FA 2:0 ratio 2.31 (3.4% coefficients of variation (CV), range 91% to 108% of the mean), FA 3:0 ratio 1.02 (2.8% CV, range 95% to 115% of the mean), FA 4:0 ratio 0.59 (5.5% CV, range 97% to 110% of mean), and FA 3:0(2Me) ratio 0.40 (4.4% CV, range 81% to 113% of the mean). Moreover, the mean of these ratios closely resembles those of the authentic standards and calibrators. For the same study, the variation of the intensities of the internal standards were calculated as a surrogate for the matrix effects, as follows: [^13^C,D_3_]-FA 2:0 (6.2% CV, range 85% to 118% of the mean), [D_5_]-FA 3:0 (5.6% CV, range 82% to 113% of the mean), and [D_7_]-FA 4:0 (3.0% CV, range 92% to 107% of the mean). In addition to the low variation in the IS intensities between the different samples, the intensities observed in the fecal samples were comparable to those in the IS blanks and low calibrator levels with diluted sample matrix samples (data not shown).

### 3.3. Calibration

Calibration lines were generated in pooled matrix samples, except for the low calibration levels, which were prepared in a diluted matrix (see Materials and Methods section). These calibrators were quantified using aqueous standard solutions. The quantification was performed using the respective matching stable isotope labelled IS. For branched chain FA 3:0(2Me), [D_7_]-FA 4:0 was utilized as IS, which showed a retention time in-between both of the C4-isomers (Figure 1). The calibration lines were linear over a wide range (Supplementary Figure S2A–D). 

### 3.4. Limit of Detection (LoD) and Reproducibility

The frequently determination of the LoD for LC-MS/MS involves a calculation of the signal to noise (S/N) ratios. Here, S/N could be applied only for FA 4:0 (LoD 0.06 µmol/g DW) and FA 3:0(2Me) (LoD 0.03 µmol/g DW), because the background peaks are virtually absent. However, for FA 2:0 and FA 3:0, the internal standard banks exhibited background signals typically with an S/N of 5 to 7. Therefore, we decided to calculate the LoD for acetate and propionate from the variation of the internal standard blanks and the variation of a sample with a low concentration (eight-fold dilution of low QC in Table 2; analysis of five sample aliquots), as described by Armbruster et al. [15] (Supplementary Table S1). Thus, the LoDs were calculated of 1.9 and 0.20 µmol/g DW for FA 2:0 and FA 3:0, respectively (Table 1).

The reproducibility of the method was tested in three different human fecal samples, with different levels of SCFA that were applied as the quality controls (QCs). For all of the QCs, the intra-day variation was below 6% (Table 2). From day to day, the variations were all below 10%.

Repeated measurement showed that analyte to IS ratio did not changed after two days in a cooled autosampler (10 °C, data not shown). Thus, accurate quantification is also possible after storage of derivatized samples.

### 3.5. SCFA Concentrations in Human Feces

The SCFAs were determined in 22 volunteers (Table 3). The mean concentrations of FA 2:0 exceeded those of FA 3:0 and FA 4:0 by about three-fold, while these are about seven-fold higher than the isobutyric acid concentrations. While the variations observed between the subjects for straight chain SCFAs were about 60% CV, the isobutyrate level displayed a lower variation with about 40% CV. Similarly, the span of concentrations were lower than five-fold for FA 3:0 (2Me) compared with more than 10-fold for the straight SCFAs. 

### 3.6. Pre-Analytic Stability in Human Fecal Samples

**Figure 2:**
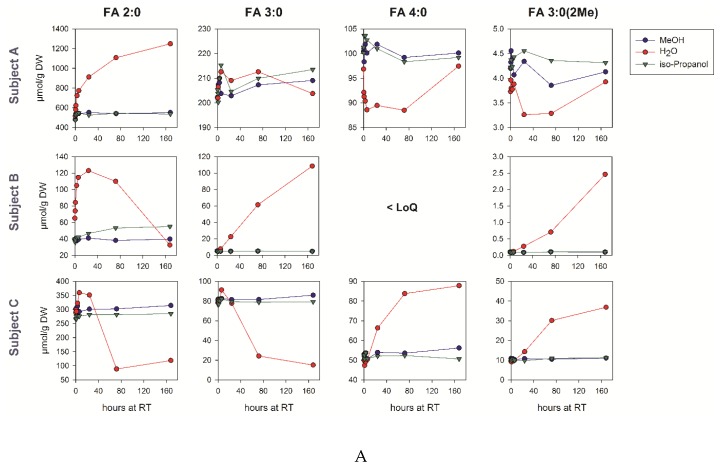

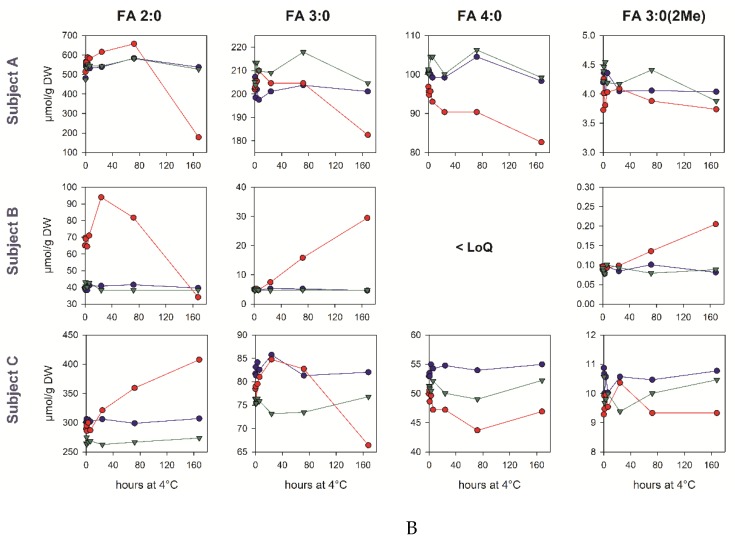
Pre-Analytic stability of SCFAs in human fecal samples. The homogenates of the fecal samples from three human donors, prepared either in water, 70% methanol, or 70% isopropanol, were kept for the indicated time at room temperature (panel **A**) or at 4 °C (panel **B**).

As microbial activity may continue after defecation, we asked whether the SCFA levels are altered by sample storage at room temperature or 4 °C. Moreover, it was tested if the potential SCFA metabolism in the fecal samples can be inhibited by the addition of organic solvents. Therefore, we homogenized the fecal samples of three volunteers within 3 h after defecation, and prepared aliquots in water, 70% methanol, and 70% isopropanol with identical concentrations. The aliquots were stored up for to 7 days and the SCFA concentrations were quantified (Figure 2). Huge differences were observed between the subjects concerning the concentrations and stability of the individual SCFAs during storage. In the aqueous samples, the SCFA concentrations, especially those of acetate in subject B, rose about two-fold within six hours. For this sample, the initial FA 2:0 concentration was ~50% higher than in the methanol/isopropanol aliquots. While the SCFA concentrations typically rise during the storage of aqueous samples, long-term storage for several days may also result in a significant drop, for example, below 50% of the initial FA 2:0 concentration after three days for subject C. These changes are substantially lower in the cold. The addition of both isopropanol and methanol stabilizes the SCFA concentrations even at room temperature.

### 3.7. SCFA in the Colon Content of Mice

**Figure 3:**
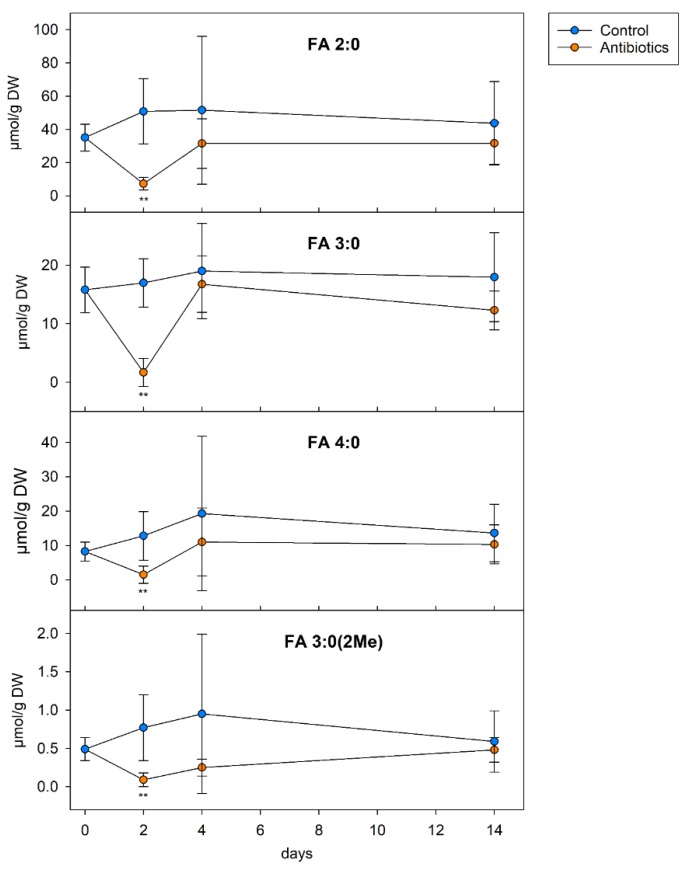
SCFA levels in the colon content of SPF mice treated with antibiotics. Specific pathogen-free mice were treated for two days with a combination of vancomycin and metronidazole (antibiotics). After treatment, the mice obtained a regular chow diet without antibiotics for an additional 2 or 12 days. The colon content was analyzed at the indicated time points (*n* = 6 each data point). For three samples with antibiotic treatment, the (day 2) FA 4:0 and FA 3:0 (2Me) concentrations were below limit of detection LoD; for these samples, two-thirds of the LoD were imputed.

In a further application of our method, we first asked whether the gut luminal SCFA levels can be linked with the microbial SCFA producers in the gut (i.e., Bacteroidetes and Firmicutes). Therefore, the mice were treated for two days with a combination of vancomycin and metronidazole (VM), before they obtained a regular chow diet without antibiotics for additional 2 or 12 days [5]. We found that the VM treatment significantly decreased the concentrations of all of the analyzed SCFAs by more than 85% compared with the controls (Figure 3). After the removal of the antibiotics, the original SCFA levels were restored. 

**Figure 4:**
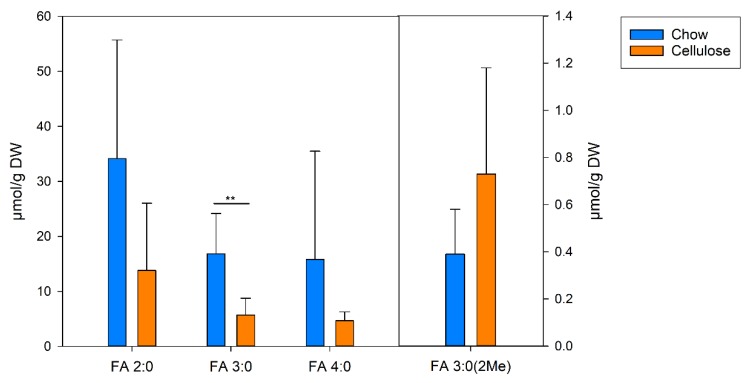
SCFA levels in the colon content of the SPF mice fed a diet containing a non-degradable and -fermentable fiber source. Specific pathogen-free mice were fed for two weeks either a chow diet containing a grain–soybean-based crude fiber extract (5%), or an experimental control diet with 5% purified cellulose instead of crude fiber. The SCFA concentrations were analyzed in the colon content (*n* = 6 each group); ** *p* < 0.01.

In the second application, we asked if the gut luminal SCFA levels can be associated with the type of dietary fiber source. The mice were fed either a chow diet containing grain–soybean-based crude fiber extract (5%), or an experimental control diet with a carbohydrate, fat, and protein content comparable to chow, but with 5% purified cellulose instead of crude fiber. Refined cellulose is practically non-degradable and non-fermentable by gut microbiota [16]. As expected, the concentrations of all of the straight chain SCFA levels dropped by more than 50% in the mice fed the cellulose-containing diet compared with the chow diet (Figure 4). Interestingly, the isobutyric acid concentration increased almost two-fold in the mice fed a non-degradable and -fermentable fiber source. However, because of the high variations, only the changes that reached FA 3:0 were of significance.

## 4. Discussion

Here, we present a fast and reproducible method for the quantification of SCFAs for up to four carbon atoms using the LC-MS/MS of 3NPH-dervatives. It is well known that accurate quantification requires the use of IS, preferably of the stable isotope labelled analyte [17]. Some methods apply labelled derivatization agents, like 13C-aniline [11] or ^13^C_6_-3NPH [9], to generate labelled SCFA derivatives. Typically, the labelled SCFA derivatives were spiked to derivatized the samples as IS [9]. Chan et al. used labelled SCFA derivatives to generate calibration lines. A drawback of such methods is that the derivatization efficiency of the individual sample could not be monitored. Here, stable isotope labelled SCFAs were added prior to the derivatization in order to control the entire sample preparation. Highly-labelled SCFAs were utilized to prevent the isotopic overlap of analyte and IS. Moreover, their high mass shift also permits application in tracer experiments using ^13^C-labeling (e.g., to trace the synthesis of fatty acids from acetate) [5,18,19]. 

Similar signal intensities of IS in the samples with and without a matrix, as well as low signal variations between the different samples, provide substantial evidence that the matrix effects are low in the present method. The specificity of the method could be demonstrated by the calculation of the ratios of the quantifier and qualifier ions. The determination of LoD for FA 2:0 and 3:0 needs consideration for their significant background levels. A similar background could also be observed for main long chain fatty acids like FA 16:0 and 18:0 [20]. However, the SCFA levels observed for both the human and mouse studies are significantly above the LoDs of the present method. The high reproducibility, at a broad range of SCFA concentrations, and the short run time of only 4 min, make this method a valuable tool for large scale studies. 

The SCFA concentrations were normalized here to the DW determined in an aliquot of the homogenized sample. It is well known that the water content of the feces may vary substantially (normal stool about 75%), especially when diarrheic samples (>85%) are included [21,22]. As we also aim for the application of this method in a disease-related context, the dilution effects need to be addressed, as shown for the application of fecal elastase in patient diagnosis [22]. Therefore, samples were adjusted to 2mg DW/mL, similar to that described for the sterol/stanol quantification [12]. As the reported SCFA concentrations in the literature are largely related to the wet weight, we multiplied these concentrations by four to get a concentration related to the DW. This factor is based on normal stools with a mean water content of 75% [21], or on data showing a median fecal wet mass production of 128 g/cap/day, with a median dry mass of 29 g/cap/day [23]. So, we recalculated the mean SCFA concentrations of the human fecal samples reported in previous studies to µmol/g DW, as follows: Han et al. [9], 6 subjects determined by LC-MS/MS (FA 2:0—138, FA 3:0—38, FA 4:0—12, FA 3:0 (2Me)—25); Hoverstad et al. [24], 20 subjects determined by vacuum distillation and subsequent gas chromatography (FA 2:0—150, FA 3:0—50, FA 4:0—50, FA 3:0 (2Me)—9); Gardana et al. [25], 40 subjects determined by LC-HRMS (FA 2:0—170, FA 3:0—68, FA 4:0—48, FA 3:0(2Me)—8); and Garcia-Villalba et al. [26], 8 subjects determined by GC-MS (FA 2:0—127, FA 3:0—28, FA 4:0—28, FA 3:0(2Me)—6). The mean concentrations observed here, were about two- to three-fold higher, which may either be related to the analyzed sample collective and/or to a bias introduced by assumption of the water content. The ratio of SCFA concentrations typically about 3:1:1:0.15 for FA 2:0/FA 3:0/FA 4:0/FA 3:0(2Me) determined in most of the previous studies fits to our data and to SCFA level detected in the large intestine [27].

To achieve accurate concentrations, pre-analytic stability of analytes needs to be evaluated [28]. This is of special importance for metabolically highly active specimen like fecal samples [29]. Gratton et al. investigated the fecal metabolome using 1H NMR spectroscopy, and recommended the transportation of samples on ice [30], because an increase in the SCFA concentrations was observed within a few hours at room temperature. Interestingly, the freezing of samples and their subsequent storage at room temperature resulted in decreased SCFA concentrations. Here, we evaluated the storage effects in three different subjects, and realized huge differences between the subjects concerning the changes during storage. Changes were smaller at lower temperatures and could be prevented by the addition of organic solvents like methanol or isopropanol. These data confirm the data of Torii et al., demonstrating that addition of 70% ethanol stabilizes fecal SCFA concentrations at room temperature [31]. As the methanol and isopropanol did not show significant differences in stabilizing the concentrations of SCFA, we prefer the addition of isopropanol directly upon the collection of stool samples. Furthermore, the addition of isopropanol to the fecal samples decreased the microbiological hazards to the laboratory staff dealing with the analysis of those samples.

Finally, the method was applied to investigate the colon content of the mice collected in a previous study investigating the effect of intestinal microbial colonization on hepatic lipid metabolism [5]. The high variation of the SCFA level between the mice may be related to the variability in the amount and distribution of the colon content. Both treatment with antibiotics and dietary intervention with a non-degradable and -fermentable fiber source resulted, as expected, in a decrease of SCFA concentrations. The finding that the isobutyric acid levels were dropped only when the mice were treated with antibiotics, but not in the cellulose-containing dietary intervention, can be explained by its generation from branched-chain amino acids, but not dietary fiber [6]. 

## 5. Conclusions

In conclusion, the presented method provides a fast and robust quantification of fecal SCFA concentrations, which represent a useful tool for both basic research and large-scale studies. Besides its short run time, the main advances compared to previous methods are the application of quantifier/qualifier ions and the matching stable isotope labelled internal standards. Moreover, it was demonstrated that the fecal SCFA level may change rapidly in aqueous samples, and the addition of organic solvents like isopropanol stops SCFA metabolism.

## Figures and Tables

**Table 1:** LC-MS/MS of the short chain fatty acid (SCFA)–3-nitrophenylhydrazones (3NPH) derivatives. Displayed are the retention time (RT), mass transitions (quantifier and qualifier ions) of analytes and stable isotope labelled internal standards, declustering potential (DP), and collision energy (CE), as well as the limit of detection (LoD) and highest calibration level for the LC-MS/MS quantification of SCFA–3NPH derivatives.

Analyt	RT (min)	Quantifier (m/z)	Qualifier (m/z)	DP (V)	CE (V)	LoD (µmol/g DW)	Highest Calibrator (µmol/g DW)
**FA 2:0**	1.18	194.1 > 151.1	194.1 > 137.1	−50	−17	1.9	1020
**[^13^C,D_3_]-FA 2:0**	1.17	198.1 > 153.1	198.1 > 137.1	−50	−17	-	-
**FA 3:0**	1.63	208.1 > 165.1	208.1 > 137.1	−60	−20	0.2	446
**[D_5_]-FA 3:0**	1.61	213.1 > 170.1	213.1 > 137.1	−60	−20	-	-
**FA 4:0**	2.53	222.1 > 179.1	222.1 > 137.1	−60	−20	0.06	440
**FA 3:0 (2Me)**	2.36	222.1 > 179.1	222.1 > 137.1	−60	−20	0.03	235
**[D_7_]-FA 4:0**	2.47	229.1 > 185.1	229.1 > 137.1	−60	−20	**-**	-

**Table 2:** Precision of SCFA–3NPH quantification. Displayed are within-run (*n* = 5) and between-run (*n* = 6) coefficients of variations (CVs) for fecal samples with low, medium, and high SCFA concentrations. Mean concentrations are in µmol/g dry weight (DW).

	FA 2:0		FA 3:0		FA 4:0		FA 3:0(2Me)	
	Mean	CV	Mean	CV	Mean	CV	Mean	CV
***intra-day (n = 5)***								
**low**	16	5.1%	4.6	6.1%	4.2	1.9%	1.3	5.1%
**medium**	203	3.8%	55	2.8%	65	1.3%	12.5	2.3%
**high**	628	2.6%	141	4.1%	122	2.1%	11.7	1.9%
***inter-day (n = 6)***								
**low**	16	3.5%	4.5	6.8%	4.1	7.5%	1.4	9.6%
**medium**	190	5.9%	53	6.5%	62	4.7%	13.8	3.5%
**high**	561	3.9%	139	4.8%	116	5.8%	12.6	4.8%

**Table 3:** SCFAs concentrations in human feces. Displayed are the SCFAs concentrations determined for the fecal samples of 22 volunteers.

SCFA (µmol/g DW)	Mean ± SD	Median	Min	Max
FA 2:0	399 ± 270	341	102	1210
FA 3:0	155 ± 97	121	19	340
FA 4:0	151 ± 95	137	18	370
FA 3:0(2Me)	22 ± 9	19	10	41

## Supplementary Materials

The following are available online. Figure S1A–D: Product ion spectra of SCFA-3NPH derivatives; Figure S2A–D: Calibration lines; Table S1: LoD determination.
