# Supplementary material for: Quantification of Fecal Short Chain Fatty Acids by Liquid Chromatography Tandem Mass Spectrometry—Investigation of Pre-Analytic Stability
Source: Biomolecules. 2019 Mar 28;9(4):121. doi: 10.3390/biom9040121 (PMC6523859; doi:10.3390/biom9040121)
Supplement: Supplementary file 1 [file biomolecules-09-00121-s001.pdf]

Supplementary Data

Quantification of Fecal Short Chain Fatty Acids by Liquid Chromatography Tandem Mass Spectrometry—Investigation of Pre-Analytic Stability

Gerhard Liebisch [1,*], Josef Ecker [2,3], Sebastian Roth [1], Sabine Schweizer [1], Veronika Öttl [1], Hans-Frieder Schött [1], Hongsup Yoon [3,4], Dirk Haller [3,4], Ernst Holler [5], Ralph Burkhardt [1] and Silke Matysik [1]

[1] Institute for Clinical Chemistry and Laboratory Medicine, University Hospital Regensburg, Franz-Josef-Strauss-Allee 11, 93053 Regensburg, Germany; sebastian.roth@ukr.de (S.R.); Schweizer.sabine@gmx.net (S.S.); Veronika.Oettl@stud.uni-regensburg.de (V.O.); hans-frieder.schoett@isas.de (H.-F.S.); Ralph.Burkhardt@ukr.de (R.B.), silke.matysik@ukr.de (S.M.)

[2] Nutritional Physiology, Technical University of Munich, 85354 Freising, Germany; josef.ecker@tum.de

[3] ZIEL-Institute for Food & Health, Technical University of Munich, 85354 Freising, Germany; Hongsup.Yoon@tum.de (H.Y.); dirk.haller@tum.de (D.H.);

[4] Chair of Nutrition and Immunology, Technical University of Munich, 85354 Freising, Germany

[5] Department of Hematology and Oncology, Internal Medicine III, University Hospital Regensburg, 93053 Regensburg, Germany; Ernst.Holler@ukr.de

* Correspondence: gerhard.liebisch@ukr.de; Tel.: +49-941-944-6240

Suppl. Figure 1A-D: Product ion spectra of SCFA-3NPH derivatives

Product ion spectra were generated in the enhanced product ion mode at a collision energy of -20 V.

FA 2:0

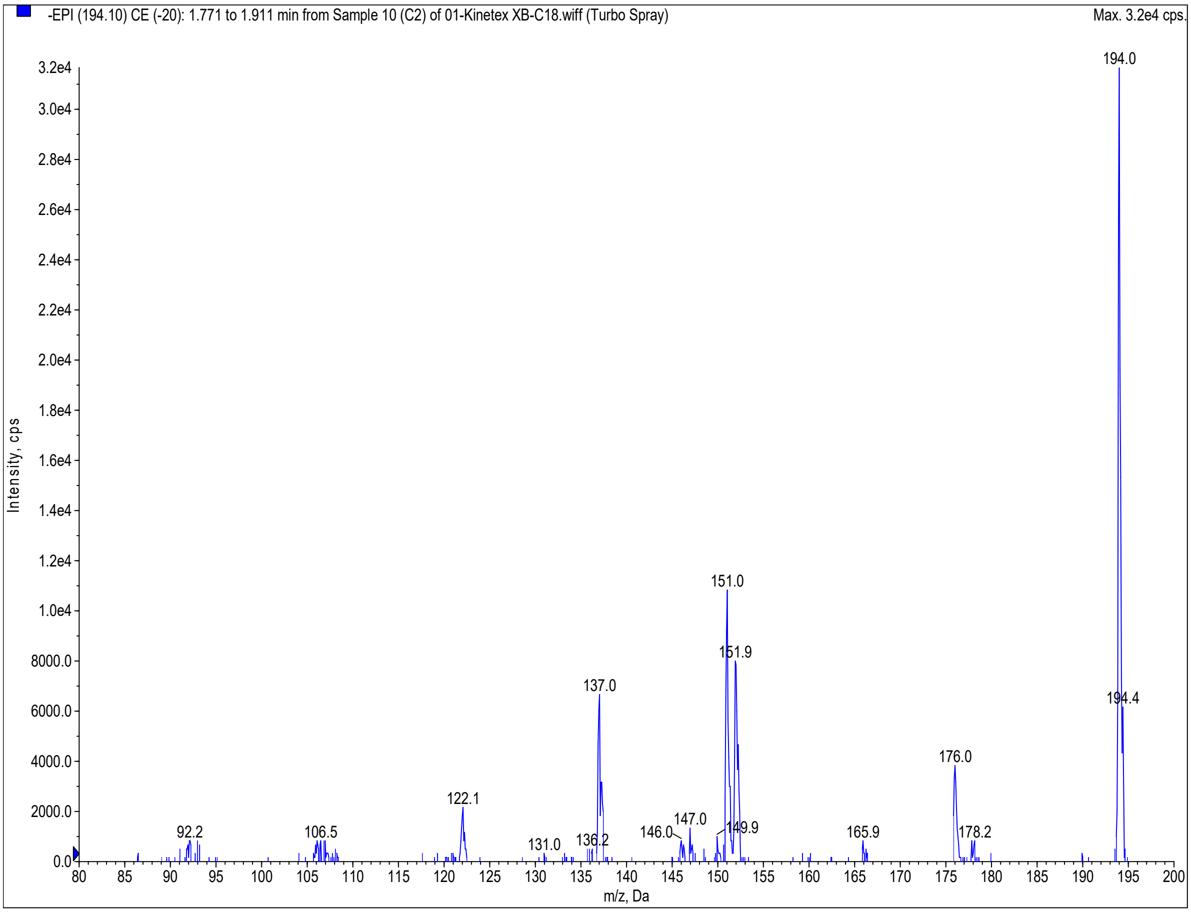

Figure S1A

FA 3:0

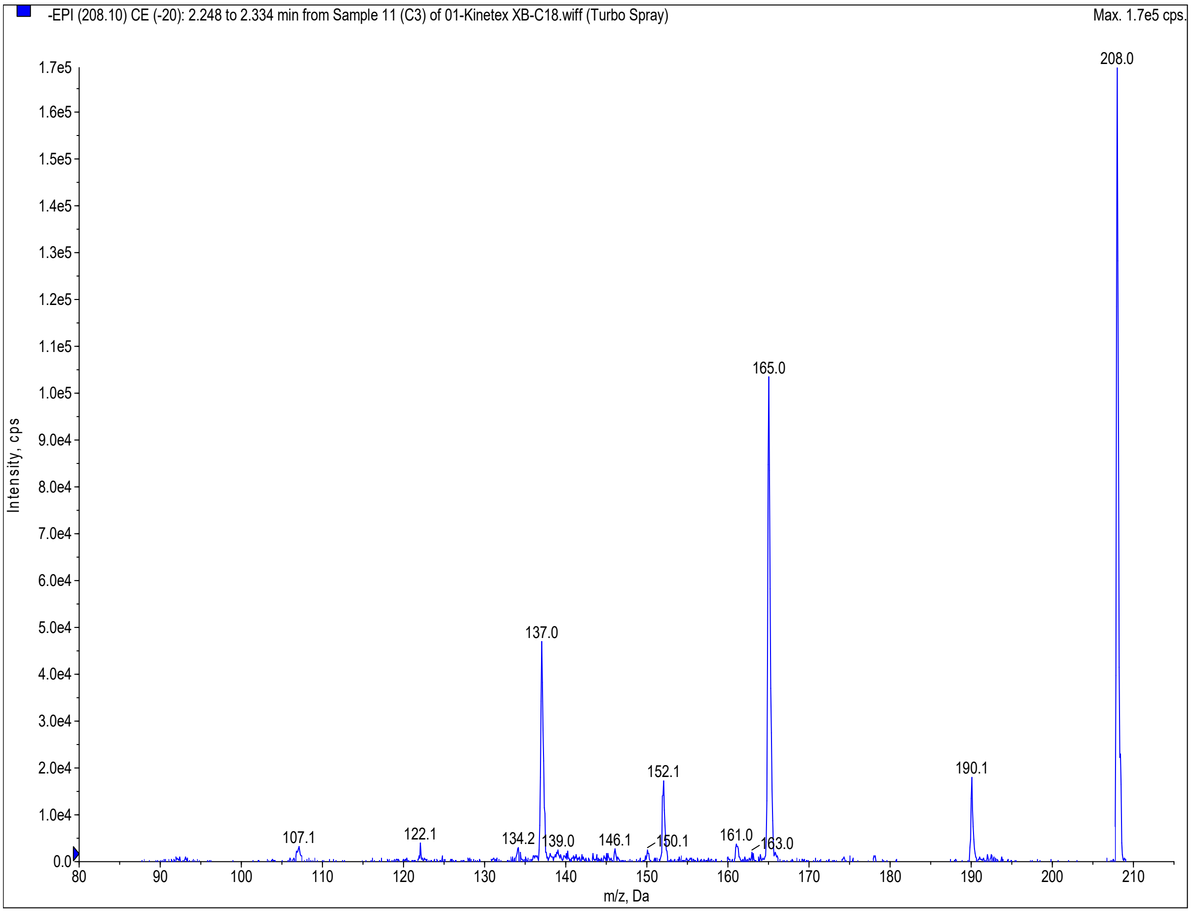

Figure S1B

FA 4:0

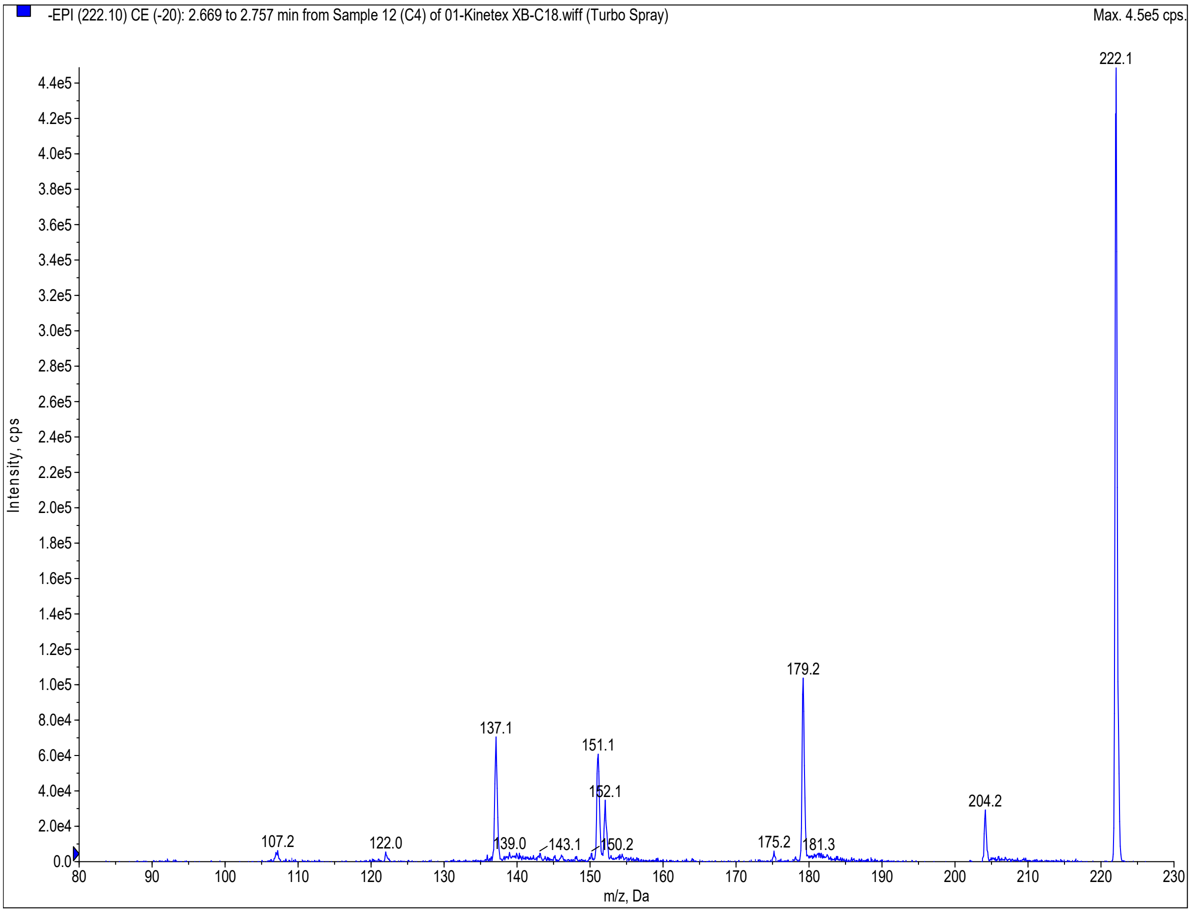

Figure S1C

FA 3:0(2Me)

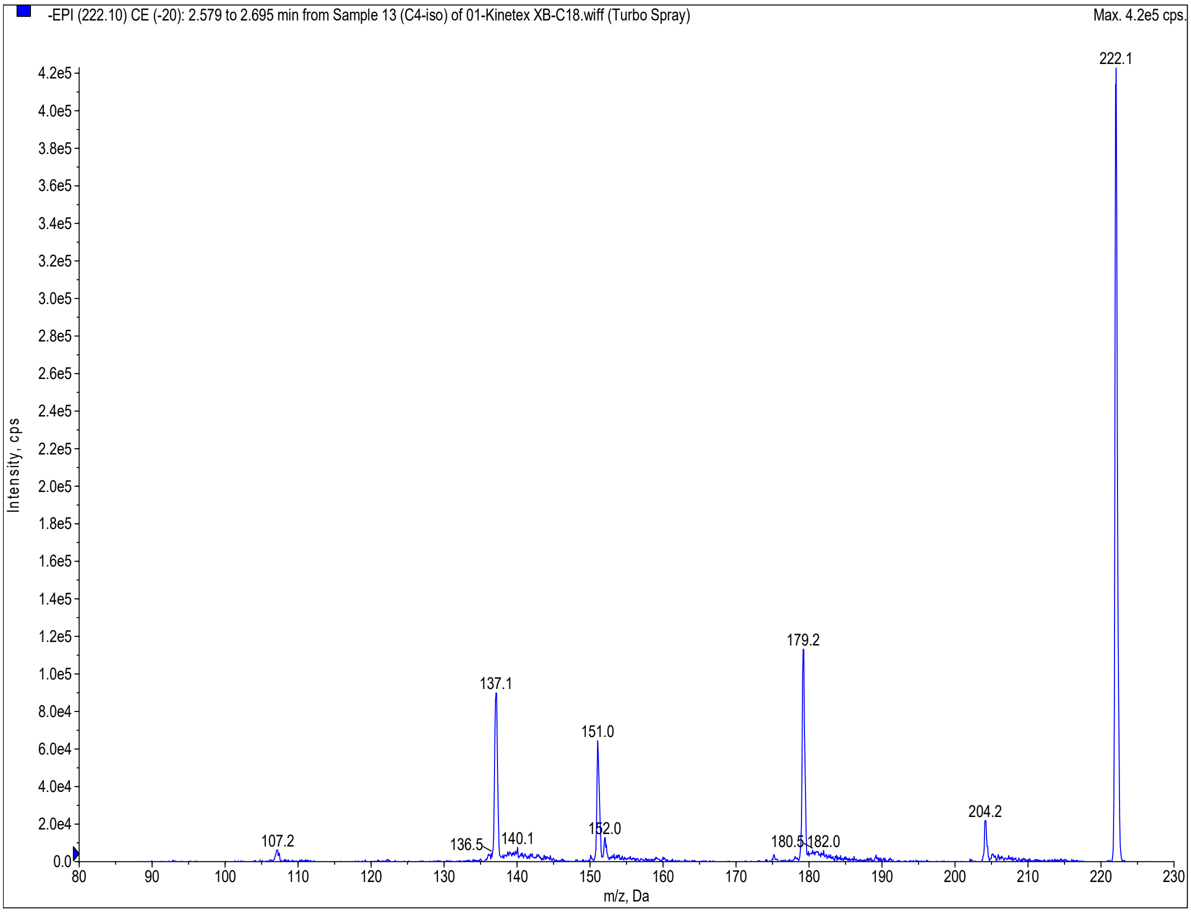

Figure S1D

Suppl. Figure 2A-D: Calibration lines:

Typical calibration line with 6 matrix based levels. Analyte concentrations are displayed in **µmol/2g DW.**

FA 2:0

	Sample Name	Sample Type	Analyte Peak Area (counts)	Analyte Concentration	IS Peak Area (counts)	Calculated Concentration	Accuracy (%)
1	CAL 1_Eigen_07-18	Standard	1.21e+004	0.461	1.32e+006	0.387	84.0
2	CAL 2_Eigen_07-18	Standard	1.27e+005	27.3	1.31e+006	31.2	114.
3	CAL 3_Eigen_07-18	Standard	2.51e+005	60.1	1.37e+006	61.4	102.
4	CAL 4_Eigen_07-18	Standard	1.12e+006	296.	1.29e+006	301.	102.
5	CAL 5_Eigen_07-18	Standard	3.48e+006	1080.	1.17e+006	1040.	97.0
6	CAL 6_Eigen_07-18	Standard	6.00e+006	2040.	1.02e+006	2060.	101.

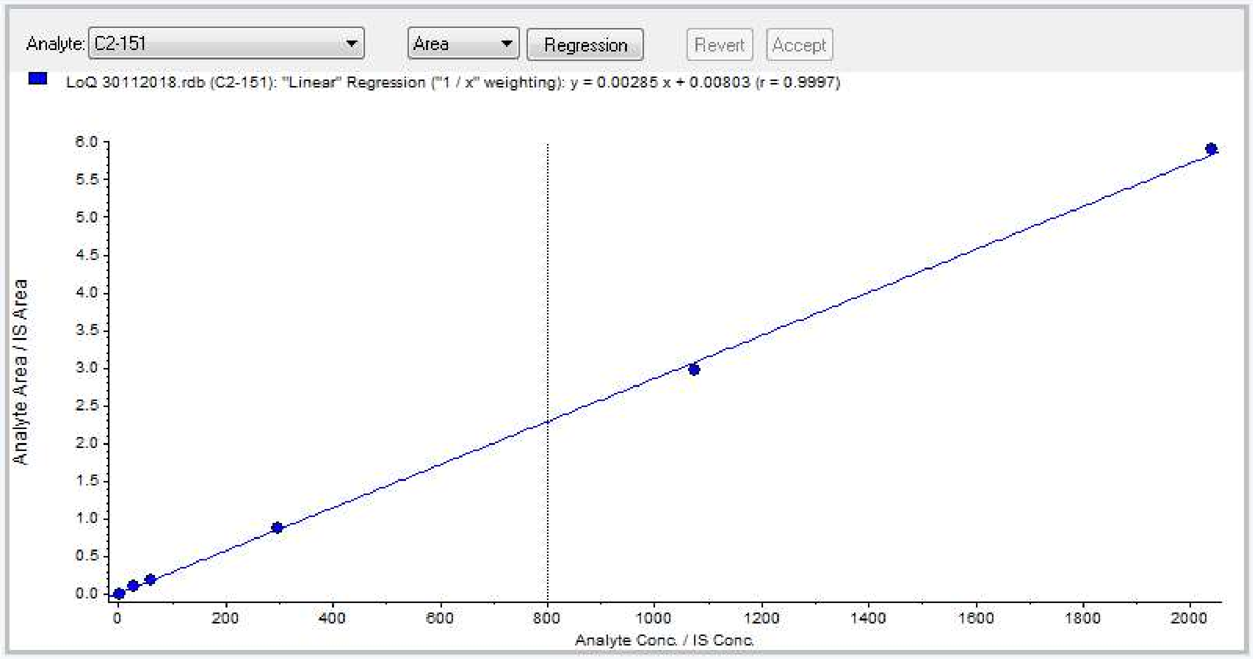

Figure S2A

FA 3:0

	Sample Name	Sample Type	Analyte Peak Area (counts)	Analyte Concentration	IS Peak Area (counts)	Calculated Concentration	Accuracy (%)
1	CAL 1_Eigen_07-18	Standard	4.96e+003	0.430	2.48e+006	0.412	95.7
2	CAL 2_Eigen_07-18	Standard	1.69e+005	9.43	2.41e+006	10.2	108.
3	CAL 3_Eigen_07-18	Standard	3.43e+005	19.9	2.61e+006	18.9	95.1
4	CAL 4_Eigen_07-18	Standard	1.55e+006	93.4	2.32e+006	95.9	103.
5	CAL 5_Eigen_07-18	Standard	6.66e+006	466.	2.08e+006	457.	98.1
6	CAL 6_Eigen_07-18	Standard	1.10e+007	893.	1.75e+006	899.	101.

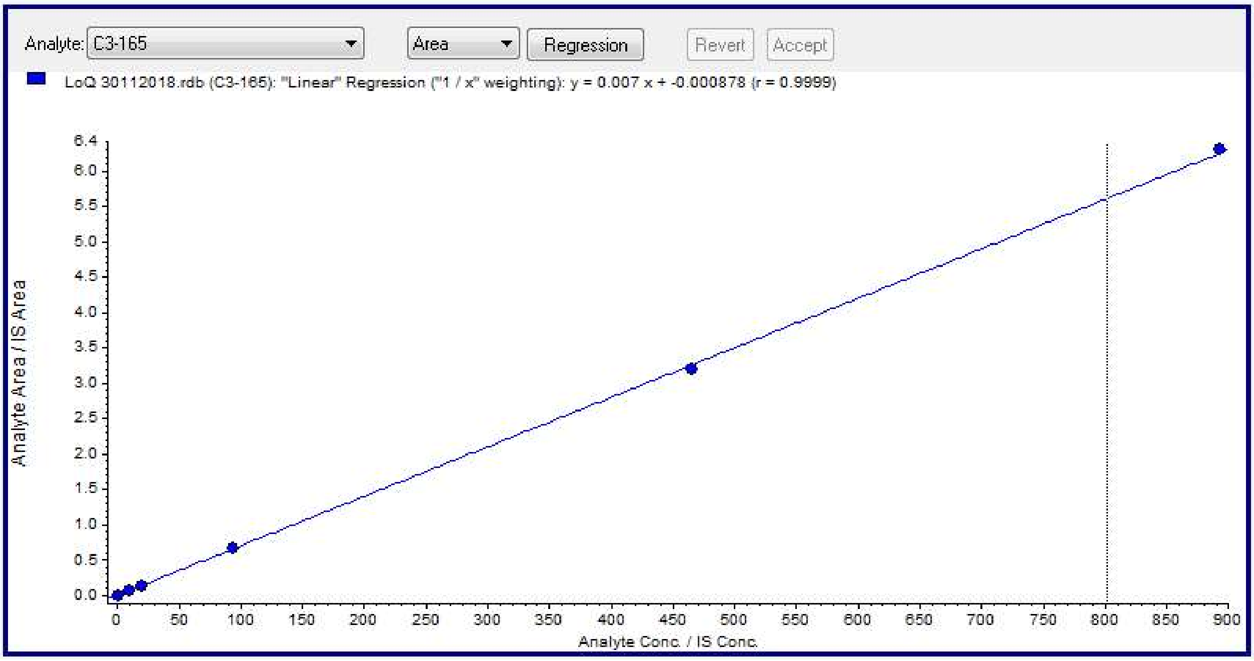

Figure S2B

FA 4:0

	Sample Name	Sample Type	Analyte Peak Area (counts)	Analyte Concentration	IS Peak Area (counts)	Calculated Concentration	Accuracy (%)
1	CAL 1_Eigen_07-18	Standard	0.00e+000	0.0819	2.59e+006	No Peak	0.00
2	CAL 2_Eigen_07-18	Standard	9.25e+004	8.53	2.54e+006	8.56	100.
3	CAL 3_Eigen_07-18	Standard	1.92e+005	17.8	2.63e+006	17.0	95.6
4	CAL 4_Eigen_07-18	Standard	9.02e+005	84.8	2.53e+006	82.7	97.5
5	CAL 5_Eigen_07-18	Standard	4.64e+006	465.	2.35e+006	459.	98.8
6	CAL 6_Eigen_07-18	Standard	7.89e+006	879.	2.06e+006	887.	101.

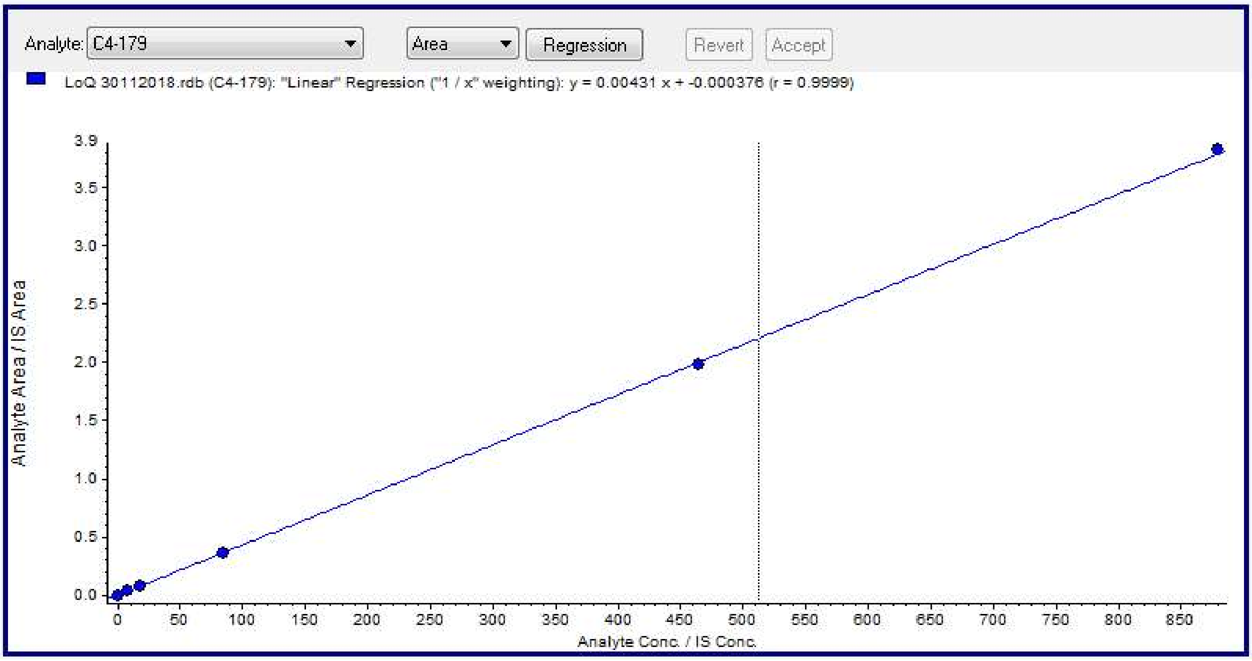

Figure S2C

FA 3:0(2Me)

	Sample Name	Sample Type	Analyte Peak Area (counts)	Analyte Concentration	IS Peak Area (counts)	Calculated Concentration	Accuracy (%)
1	CAL 1_Eigen_07-18	Standard	0.00e+000	0.0761	2.59e+006	No Peak	0.00
2	CAL 2_Eigen_07-18	Standard	7.89e+004	5.99	2.54e+006	6.04	101.
3	CAL 3_Eigen_07-18	Standard	1.65e+005	12.9	2.63e+006	12.1	93.5
4	CAL 4_Eigen_07-18	Standard	7.56e+005	60.7	2.53e+006	57.2	94.2
5	CAL 5_Eigen_07-18	Standard	3.00e+006	256.	2.35e+006	245.	95.7
6	CAL 6_Eigen_07-18	Standard	5.24e+006	471.	2.06e+006	486.	103.

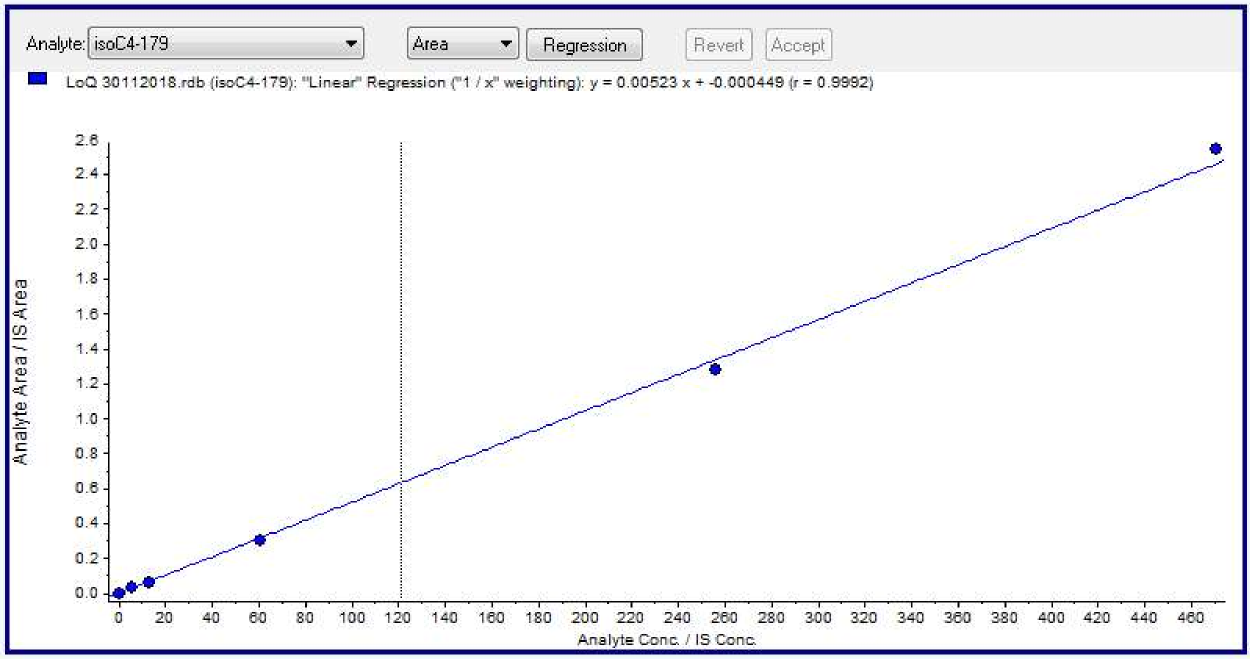

Figure S2D

Suppl. Table 1: LoD determination

	FA 2:0	FA 3:0
**Mean (blank)**	1.06	0.10
**SD (blank)**	0.23	0.03
**LoB**	1.44	0.15
**SD (low conc.)**	0.27	0.028
**LoD**	1.88	0.20

LoB = mean(blank) + 1.645(SD blank)

LoD = LoB + 1.645(SD low concentration sample)

Displayed are mean and standard deviation calculated from internal standard blanks of nine different batches. Variations at low concentrations were calculated from the analysis of five sample aliquots of an 8-fold dilution of low QC (see Table 2). Limit of blank (LoB) and limit of detection (LoD) are calculated using the displayed equations. Values are [µmol/g DW]
